# Data on performance prediction for cloud service selection

**DOI:** 10.1016/j.dib.2018.08.108

**Published:** 2018-08-31

**Authors:** Abdullah Mohammed Al-Faifi, Biao Song, Mohammad Mehedi Hassan, Atif Alamri, Abdu Gumaei

**Affiliations:** College of Computer and Information Science, King Saud University, Riyadh, Saudi Arabia

**Keywords:** Performance metrics, Workload parameters, Cloud computing

## Abstract

This paper contains data on Performance Prediction for Cloud Service Selection. To measure the performance metrics of any system you need to analyze the features that affect these performance, these features are called " workload parameters". The data described here is collected from the KSA Ministry of Finance that contains 28,147 instances from 13 cloud nodes. It was recorded during the period from March 1, 2016, to February 20, 2017, in continuous time slots. In this article we selected 9 workload parameters: Number of Jobs in a Minute, Number of Jobs in 5 min, Number of Jobs in 15 min, Memory Capacity, Disk Capacity,: Number of CPU Cores, CPU Speed per Core, Average Receive for Network Bandwidth in Kbps and Average Transmit for Network Bandwidth in Kbps. Moreover, we selected 3 performance metrics: Memory utilization, CPU utilization and response time in milliseconds. This data article is related to the research article titled "An Automated Performance Prediction Model for Cloud Service Selection from Smart Data” (Al-Faifi et al., 2018) [1].

**Specifications Table**

**Table:** 

Subject area	*Computer Science*
More specific subject area	*Performance prediction, cloud computing*
Type of data	*Tables*
How data was acquired	*Data was collected from the KSA Ministry of Finance that contains 28,147 instances from 13 cloud nodes. It was recorded during the period from March 1, 2016, to February 20, 2017, in continuous time slots. It is collected using manage engine (application manager) and solar winds (virtualization manager software).*
Data format	*Raw data with class labels*
Experimental factors	*A set of attributes include the number of Jobs in a Minute, number of Jobs in 5* *min, a number of Jobs in 15* *min, memory capacity, disk capacity, number of CPU cores, CPU speed per core, average receive for network bandwidth in Kbps, and average transmit for network bandwidth in Kbps. A set of predictors are memory utilization, CPU utilization and response time.*
Experimental features	*The experiment aims to build two prediction models. The first model is used to learn from labeled workload attributes and predict memory utilization, CPU utilization, and response time. The data set used in this model contains 28,147 instances. A random subset of 2450 instances is utilized as a testing set. The second model is used to learn from CPU utilization and response time of one model type node as a benchmark and predict CPU utilization and response time of another model type node.*
Data source location	*Ministry of Finance, Riyadh, Saudi Arabia*
Data accessibility	*Data is available with this article*

**Value of the data**

• This dataset is important to the field of performance prediction and cloud computing as it provides a log of workload parameters as well as performance metrics.
• The data could be used as a benchmark for performance prediction.
• Analysis of the data can provide direction towards enhancing the performance of the systems and helping in identifying the resources required before in migrating to cloud service.

## 1 Data

The supplementary dataset contains 28,147 instances from 13 cloud nodes. These data was recorded during the period from March 1, 2016, to February 20, 2017, in continuous time slots. These data contains nine workload parameters include: Number of Jobs in a Minute, Number of Jobs in 5 min, Number of Jobs in 15 min, Memory Capacity, Disk Capacity,: Number of CPU Cores, CPU Speed per Core, Average Receive for Network Bandwidth in Kbps and Average Transmit for Network Bandwidth in Kbps. Other than that, three performance metrics were selected include: Memory utilization, CPU utilization and response time in milliseconds. Details of the testing scenario can be found in section 4 in [1].

The supplementary files contains all 28,147 instances of both workloads and performance metrics.

## 2 Experimental design, materials and methods

### 2.1 Dataset collection

We collected a large workload dataset from the KSA Ministry of Finance that contains 28,147 instances from 13 cloud nodes. It was recorded during the period from March 1, 2016, to February 20, 2017, in continuous time slots. These different date periods of collecting the data provided more diversity to allow a fair test of the classifier and more accurate evaluation of the work. In the model, nodes 1 and 5 are HP RP 4440, nodes 2–4 and 6 are HP RP 7420, and nodes 7–13 are HP DL 380 G5. The number of instances collected for some nodes may differ because they were out of service during the data recording phase. Therefore, we gathered 2427 instances from node 1, 2426 instances from nodes 2–5, 2232 instances from nodes 6 and 8–13, and 392 instances from node 7. A description of the dataset is shown in the following Table 1:

• Attributes information:

1) F1: Number of Jobs in a Minute.
2) F2: Number of Jobs in 5 min.
3) F3: Number of Jobs in 15 min.
4) F4: Memory Capacity.
5) F5: Disk Capacity.
6) F6: Number of CPU Cores.
7) F7: CPU Speed per Core.
8) F8: Average Receive for Network Bandwidth in Kbps.
9) F9: Average Transmit for Network Bandwidth in Kbps.

• Responses information:

1) R1: Memory Utilization in percent.
2) R2: CPU Utilization in percent.
3) R3: Response Time in milliseconds.

• Responses of our dataset are converted into four categorical class labels as follows:
  ■ Very Low for data between 0% and 25%.
  ■ Low for data between 26% and 50%.
  ■ Medium for data between 51% and 75%.
  ■ High for data between 75% and 100%.

**Table 1:** Data set description.

**Data set characteristics:**	Multivariate	**Number of instances:**	28,147	**Area:**	Computer
**Attribute Characteristics:**	Real	**Number of Attributes and Responses:**	12	**Date Donated**	2017-06-01
**Associated Tasks:**	Classification, Regression	**Missing Values?**	No	**Number of nodes and Model Types**	13 nodes. Nodes 1 and 5 are HP RP 4440. Nodes 2–4 and 6 are HP RP 7420. Nodes 7–13 are HP DL 380 G5

### 2.2 Method and results

We used a Naïve Bayes (NB) with kernel density estimation (KDE) classifier in two prediction models. The first model has been used to learn from labeled workload attributes (F1–F9) and predict memory utilization (R1), CPU utilization (R2), and response time (R3) from unlabeled workload attributes (F1–F9). A data set used in the first model contains 28,147 instances. A random subset of 2450 instances is used as the testing set to test the classifier for accuracy and the rest of those instances are used as the training set to build the classifier.

The second model has been used to learn from labeled CPU utilization (R2) and response time (R3) of one model type node as a benchmark and predict CPU utilization (R2) and response time (R3) from unlabeled CPU utilization (R2) and response time (R3) of another model type node.

For training and testing of the second model, we used the data instances of one node from HP RP 4440 model type as a benchmark for training the Naïve Bayes classifier and the data instances of another node from HP RP 7420 model type for testing the Naïve Bayes classifier. Here, we used a data of node 1 from HP RP 4440 model type which contains 2427 instances as a benchmark for training and data of node 2 from HP RP 7420 model type for testing. The goal of second model is to predict the CPU utilization (R2) and response time (R3) if we run the same jobs in two different model types of nodes.

**Fig. 1:**
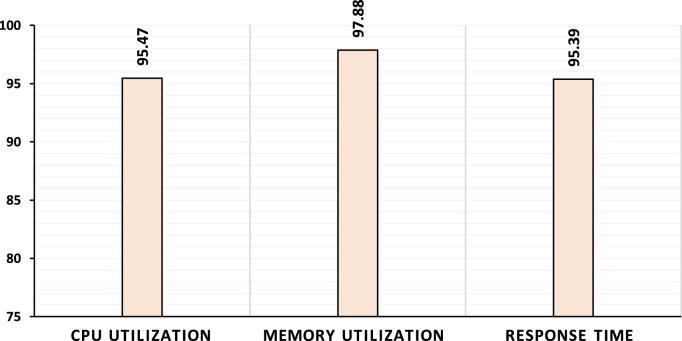
Prediction rates of first model.

**Fig. 2:**
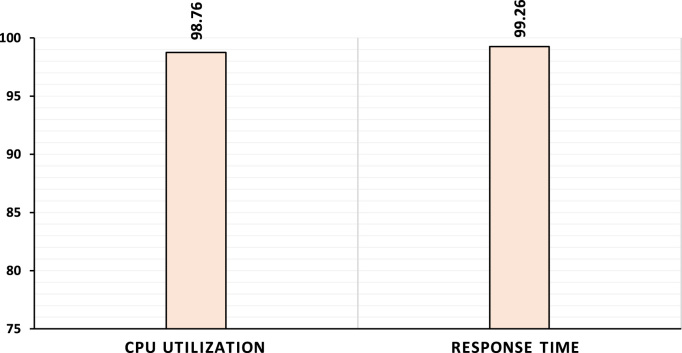
Prediction rates of second model.

Using the first model, we achieved accuracy rates up to 95.47%, 97.88% and 95.39% for CPU utilization (R2), Memory utilization (R1) and Response time (R3), respectively, as shown in Fig. 1. Additionally, in Fig. 2, we achieved accuracy rates up to 98.76% and 99.26% by using the second model with respect to the CPU utilization (R2) and response time (R3).
